# Investigating the impact of anthropogenic noise on the decision-making of dwarf mongoose offspring

**DOI:** 10.1098/rsos.240192

**Published:** 2024-05-22

**Authors:** Lauren S. Vane, Amy Morris-Drake, Josh J. Arbon, Robyn J. Thomson, Megan Layton, Julie M. Kern, Andrew N. Radford

**Affiliations:** ^1^ School of Biological Sciences, University of Bristol, Bristol BS8 1TQ, UK; ^2^ School of Environmental and Rural Science, University of New England, Armidale NSW 2351, Australia

**Keywords:** anthropogenic noise, social behaviour, acoustic communication

## Abstract

Anthropogenic (man-made) noise constitutes a novel and widespread pollutant which is increasing in prevalence in terrestrial and aquatic ecosystems, resulting in alterations of natural soundscapes. There is proliferating evidence that noise leads to maladaptive behaviour in wildlife, yet few studies have addressed the effect on mammalian parent–offspring interactions. We investigated the impact of road noise on dwarf mongoose (*Helogale parvula*) offspring nearest-neighbour decision-making while foraging, using a field-based playback experiment. We predicted that offspring would forage closer to groupmates, especially adult and dominant individuals, when experiencing road noise compared with ambient sound to reduce communication masking and alleviate stress. We also predicted that noise would have a reduced effect with increasing offspring age owing to reduced reliance on adult groupmates for provisioning and predator defence. However, we found that mean nearest-neighbour distance and nearest-neighbour intrinsic characteristics (age, sex and dominance status) did not differ significantly between sound treatments, and these responses did not vary significantly with focal individual age. Noise may not impact nearest-neighbour decision-making owing to habituation from chronic natural exposure; alternatively, noise could induce stress and distraction, resulting in maladaptive decision-making. Future work should aim to detangle the underlying mechanisms mediating parent–offspring interactions in conditions of anthropogenic noise.

## 1. Introduction

Anthropogenic (man-made) noise constitutes a novel and prevalent pollutant to which wildlife communities are becoming increasingly exposed. While anthropogenic noise (hereafter ‘noise’) can be intentional, such as that generated by sonar or seismic testing, it is often a byproduct of activities associated with human population growth, including urbanization, transportation and resource extraction [1,2]. Noise differs in acoustic structure, frequency and amplitude from naturally occurring ambient sound [3], resulting in the alteration of natural soundscapes [4,5]. There is a growing body of evidence documenting the adverse effects of noise on a wide array of taxa [1,5–7]. Noise can have physiological and developmental consequences [8,9] or cause changes to the distribution and abundance of populations [10]. Behavioural responses can occur at the lowest levels of noise, including far from the source, and are particularly important because they represent the first line of defence for organisms in a changing world [11]. Hence, behavioural responses are the most prevalent and extensively studied consequence of noise [2].

Noise induces changes in behaviour via four mechanisms which are not mutually exclusive. First, animals may perceive noise as a direct threat, eliciting costly avoidance behaviours equivalent to those seen in a predatory context [12]. Second, noise can induce stress, resulting in inappropriate physiological and behavioural responses [13,14]. Third, noise may distract an individual’s finite attention away from tasks such as sending or receiving signals, foraging or predator avoidance [15,16]. Lastly, noise can mask biologically relevant acoustic information; masking is the increase in the threshold for detection of a signal or cue owing to noise at overlapping frequencies [17–19]. There is extensive experimental evidence for the detrimental behavioural consequences of noise to individuals; for example, noise reduces foraging efficiency in fish [20], birds [21] and mammals [22,23], and reduces the efficacy of predator-avoidance strategies in a range of species [24–27]. In social species, noise is likely to be encountered by multiple individuals simultaneously, so can affect interactions between them [28,29]. Within-species interactions are often regulated by acoustic signals, and can thus be disrupted by noise [24,30]. Some signallers therefore alter their acoustic signals, such as by altering the structure, amplitude or frequency of vocalizations, to enhance transmission when it is noisy [6,17,31]. Interactions between parents and offspring, particularly relating to parental care, are crucial for fitness. Evidence that noise negatively affects parent–offspring interactions in avian species, hindering communication [32] and altering parental-care behaviour [33], has previously only considered adult responses to noise. To our knowledge, the influence of noise on mammalian parent–offspring interactions, particularly, how noise may impact offspring decision-making, has rarely been investigated (for an exception, e.g. [34]).

It is increasingly apparent that there is considerable interspecific and intraspecific variation in behavioural responses to noise [8,35]. Among members of the same species, some of this variation results from extrinsic factors, which can alter the context in which noise is encountered; for example, habitat cover [36], the presence of conspecifics [37] and the existence of other stressors [38]. Individual characteristics, such as dominance status [38], body condition [39], experience [40] and sex [41], can also drive variation in responses to noise. Younger individuals may be more responsive to noise owing to their lesser experience and higher predation risk compared with adults [42,43]; higher predation risk means that it is beneficial to be more responsive to cues indicative of a predation threat but increases the risk of misclassifying a non-predatory signal, such as noise, as predatory [44–47]. Previous work on age-related differences in response to noise has found younger individuals to be more noise-avoidant than adults in birds [48] and mammals [38]. We know much less about age-dependent variation in other behavioural responses to noise.

Dwarf mongooses (*Helogale parvula*) provide a valuable opportunity to investigate the impact of noise on adult–offspring interactions. They are vocal, carnivorous, diurnal mammals which live in cooperatively breeding groups of up to 30 individuals [49]. Groups contain a dominant breeding pair and non-breeding subordinates of both sexes [50]. Dominant pairs produce litters at regular intervals, usually three times per breeding season [49,50]. Litters remain in the breeding burrow for 10–14 days post-birth; after first emerging, they stay close to the burrow (protected by ‘babysitters’) for about a fortnight. Offspring begin accompanying the group during foraging excursions at around 3–4 weeks of age, maintaining close proximity and with regular provisioning of prey items from all older group members [51,52]. When an adult finds a prey item, it often produces a specific ‘provisioning vocalization’ to encourage offspring to approach and collect the food [52]. Provisioning by adults continues until offspring are around 4 months old, although independent prey capture usually begins from 6 weeks old [52]. While offspring greater than 4 months old (classified as juveniles) are likely to be foraging more independently compared with pups (<4 months old), offspring of all ages are at risk from both terrestrial and aerial predators [53] and are dependent on older group members for predator defence. Adult sentinels (raised guards) monitor threats [54,55] and subsequently alert other group members using a range of alarm calls [56]. Dominant group members are perceived as higher-quality sentinels than subordinates, with group members investing less time in personal vigilance when the former are scanning for danger [57]. Adult dwarf mongooses exhibit preferences over nearest neighbours while foraging, which may be owing to the improved social acquisition of information regarding foraging opportunities and predators [57,58]. Additionally, studies of other species have shown that stressors induce greater group cohesion and the close presence of bonded individuals reduces stress, hence such nearest-neighbour preferences exhibited in dwarf mongooses may also function for stress alleviation [59,60]. Previous experimental playbacks have shown that noise disrupts the vocal transmission of alarm calls from sentinels, and both foragers and offspring respond more strongly to noise than non-foragers and adults, respectively [24,34].

Here, we used a field-based playback experiment to investigate the impact of acute road noise on the nearest-neighbour decision-making of dwarf mongoose offspring while foraging. As adults are occupied with foraging for mobile and sporadically located food items, it is likely that the onus is on the offspring to regulate nearest-neighbour distance and identity. We predicted that during playback of road noise (compared with ambient sound), mean nearest-neighbour distances to the closest conspecific would decrease to compensate for acoustic communication masking and to alleviate noise-induced stress. We also predicted that noise would be associated with more time spent in closest proximity to adult and dominant nearest neighbours, as opposed to other offspring or subordinates, owing to the likelihood of improved acquisition of social information regarding predation risk. Lastly, we predicted that noise would have a reduced effect on juveniles compared with pups, as juveniles forage more independently and are likely to be less naive to predation risk and hence rely less on adult group members for provisioning of food and predator defence.

## 2. Methods

### 2.1. Study site and population

This study was conducted as part of the long-term Dwarf Mongoose Research Project on Sorabi Rock Lodge Reserve, a 4 km^2^ private game reserve situated in Limpopo Province, South Africa (24°11′ S, 30°46′ E). A frequently used tar road (R530) runs adjacent to the study area, meaning that road noise is an ecologically relevant pollutant [24]. The study population at the time of this work comprised six dwarf mongoose groups, habituated to human observers (<5 m proximity on foot). The longevity of the monitoring project (established in 2011) means that extensive life-history data were available, including the age, sex and dominance status of the study individuals. Individuals were identifiable via characteristic small blonde dye marks on their fur, which observers added with an elongated paintbrush. Sex was determined by observations of ano-genital grooming; the dominance status of adults (individuals >1 year)—dominant (the breeding pair) or subordinate (non-breeding adults)—was deduced from observations of feeding displacements, aggression and scent-marking behaviour [49,55]. Work was conducted under permission from the Limpopo Department of Economic Development, Environment and Tourism (permit number: 001-CPM403-00013), and ethical approval from the University of Pretoria, South Africa (Animal Ethics Committee: NAS321/2022) and the University of Bristol, UK (Animal Welfare and Ethics Review Body: UIN/17/074).

### 2.2. Field experiment

To investigate the impact of anthropogenic noise on dwarf mongoose offspring nearest-neighbour decision-making while foraging, 17 individuals (nine pups and eight juveniles) received two playback treatments: ambient sound and road noise. This sample size exceeds that of previous studies which have yielded significant effects of road-noise playback on dwarf mongooses [61,62]. We used ambient sound as a control, as opposed to white noise, for ecological validity and in line with previous work on various species [62–64]; logistical constraints associated with fieldwork meant that only one control treatment was used. We ran experimental trials in March–April 2023 on individuals belonging to three wild dwarf mongoose groups (mean ± s.d. group size: 14.3 ± 4.7, range: 9–19 individuals); other groups within the study population did not include individuals of an appropriate age. While we defined pups as individuals <4 months old and juveniles as those >4 months old, the natural interval between litters meant that all our focal pups were <3.3 months old and all juveniles were >5.3 months old at the time of their first experimental trial. No individuals aged sufficiently to mean that they moved out of their initial age class during the experimental period.

#### 2.2.1. Acoustic recordings and playback track construction

We collected all acoustic recordings using our standard protocols [24,34] with a Tascam DR100 MKIII professional audio recorder and a handheld Sennheiser ME66 shotgun microphone with a Rycote Softie windshield. While the International Organization for Standardization states that sound level meters should be positioned 1.2–1.5 m from the ground (ISO 10052), this height is biologically irrelevant to an organism which perceives sound at a height of ~10 cm. Hence, the microphone was positioned ~10 cm from the ground to mimic the height of a foraging dwarf mongoose head. We recorded ambient sound from the centre of each group’s territory at 8.00 and 16.00, in the absence of nearby dwarf mongoose groups or anthropogenic noise (such as from nearby air or road traffic). Road noise was recorded in 3 × 1 h sessions, 10 m from the main road adjacent to the study site with no physical obstructions between the road and the microphone. All recordings were collected in dry, calm conditions (no rain or wind stronger than a light breeze). A HandyMAN TEK 1345 sound-level meter was used to record peak sound–pressure amplitudes during ambient-sound and road-noise recordings at 5 m.

We used Audacity 3.2.5 to construct a unique road-noise track and an ambient-sound track for every focal individual (*n =* 17). Recordings with good sound-to-noise ratio were selected, and we removed any incongruous sounds (such as heterospecific alarm calls). Ambient-sound tracks were each 5 min in duration. Ambient trials conducted in the morning used ambient sound recorded at 8.00 and trials conducted in the afternoon used recordings taken at 16.00 to account for diurnal fluctuations in the soundscape. Road-noise tracks (also 5 min in duration) included three vehicle passes, matching the mean frequency and composition of vehicles observed on the R530 from 10 × 1 h traffic counts [24], with a randomized order of vehicle passes between tracks.

#### 2.2.2. Experimental protocol

Each focal individual received both treatments (ambient sound and road noise) in a counterbalanced order, with the treatment order for the first focal individual alternating between groups. Where possible, we completed both treatments on a single focal individual on the same day within the same session (morning or afternoon), with a minimum of 30 min between trials on the same focal individual. If both treatments were not possible on the same day, the second one was run in a matched-time session as soon as conditions allowed (mean ± s.d. time between paired trials: 1.1 ± 2.9 days, range: 1–12 days). Group composition was consistent between each paired trial and the same observer conducted both trials with the same focal individual. If babysitters (group members left at the burrow with young pups while the rest of the group foraged elsewhere) were absent during one trial, we ensured this was consistent with the paired trial. We occasionally conducted trials with different focal individuals in the same group in the same session to capitalize on appropriate sampling conditions, but a minimum of 15 min between trials was maintained between different treatment types and a minimum of 30 min was maintained between treatments of the same type to avoid habituation to playback tracks. We conducted trials when there had been no natural heterospecific or conspecific alarm call for 10 min and no major disturbances (such as an intergroup encounter or snake mob) for at least 30 min. Trials were run when the entire group was foraging at least 100 m from the nearest road in an open–medium habitat (0–66% vegetation density) under dry, calm conditions (no rain or wind stronger than a light breeze).

We broadcast all playbacks using an iPhone 12 connected via an auxiliary cable to a Rokono BASS+ mini loudspeaker (frequency response: 90–20 000 Hz). The loudspeaker was held by the observer at a height of 1 m (the approximate height of a car engine) while moving parallel to the focal individual; the observer remained 3–5 m from the focal individual throughout the trial. Prior to trials, we used a HandyMAN TEK 1345 sound-level meter to ensure peak playback amplitude during vehicle passes was 75 dB for road-noise tracks and 40–45 dB for ambient-sound tracks at 5 m, in line with natural sound levels. Playback treatments lasted a minimum of 2 min and a maximum of 5 min. We abandoned trials if, during the first 2 min, a heterospecific or conspecific alarm call occurred, the focal individual or their nearest neighbour ceased to forage (such as for sentinel duty or to interact socially), or the focal individual went out of sight and did not reappear within 30 s or was over 5 m from the loudspeaker. Abandoned trials were repeated during the same session after a minimum of 30 min or when conditions allowed in a matched-time session within 14 days.

Prior to the start of playback in a trial, an observer dictated onto a Sony ICD-PX470 digital voice recorder the focal individual identity, identity of their nearest neighbour and distance from their nearest neighbour. We recorded distances to the nearest 0.5 m up to 8 m, and then as >8 m. Following a vocal indication of the point at which playback commenced, we recorded the immediate response (within first 10 s) as none, head-up or fleeing. For the duration of the playback trial, we recorded any changes to nearest-neighbour identity and nearest-neighbour distance. We planned to record instances of provisioning to focal individuals, but there were none during the trial periods. We extracted nearest-neighbour identity and neighbour-distance at 10 s intervals during post-trial analysis of voice recordings.

### 2.3. Statistical analyses

We conducted all analyses using R version 4.3.0 [65]. Tests were two-tailed and considered significant at *p <* 0.05. We examined residuals visually, using parametric tests if the associated assumptions (normality of error, homogeneity of variance and absence of multicollinearity) were satisfied, and non-parametric tests otherwise.

We used a Wilcoxon signed-rank test to confirm that there was no significant difference in trial duration between playback of ambient-sound or road-noise treatments (*V* = 70, *n* = 17, *p* = 0.59), enabling use of the entire trial for analyses regardless of duration. We used McNemar tests to investigate immediate behavioural responses (none or head-up; there were no instances of fleeing) and changes in nearest-neighbour identity (change or no change) within 10 s of playback initiation.

We ran linear mixed-effects models (LMMs), using the lme4 package [66], to investigate the effect of playback treatment on (i) nearest-neighbour distance and (ii) nearest-adult-neighbour distance (excluding nearest neighbours which were juveniles or pups), using mean values from the entirety of each trial. We ran generalized linear mixed models (GLMMs) with beta distributions and logit link functions, using the glmmTMB package [67], to investigate the influence of intrinsic characteristics (i.e. age class, sex and dominance status) on nearest-neighbour decision-making between playback treatments. Specifically, separate GLMMs were run to consider the proportion of time per trial where the nearest neighbours were (i) adults (rather than non-adults), (ii) adult females (rather than adult males), or (iii) dominant adults (rather than subordinate adults). To enable the use of beta distribution in GLMMs, exact zero values were converted to 0.0001, and values of exactly 1 were converted to 0.9999. Such changes likely had little effect but would, if anything, make our results more conservative. In all LMMs and GLMMs, playback treatment (ambient or road), age (pup or juvenile) and their interaction were included as fixed terms, with focal individual identity nested within group membership included as a random effect to account for paired data points from each focal individual, with overlapping group membership. The significance of main effects and interactions was evaluated using the package lmerTest [68], which conducts *F*-tests using Satterthwaite’s method for calculating denominator degrees of freedom [69]. If the singular model fit was obtained, the ‘blmer/bglmer’ wrapper functions from the blme package were used [70], whereby the default Wishart covariance prior was used to provide a weakly informative prior to aid model fitting. In these cases, significance values were obtained using a comparison of the term of interest to a null model (likelihood ratio test) [71].

## 3. Results

**Figure 1.**
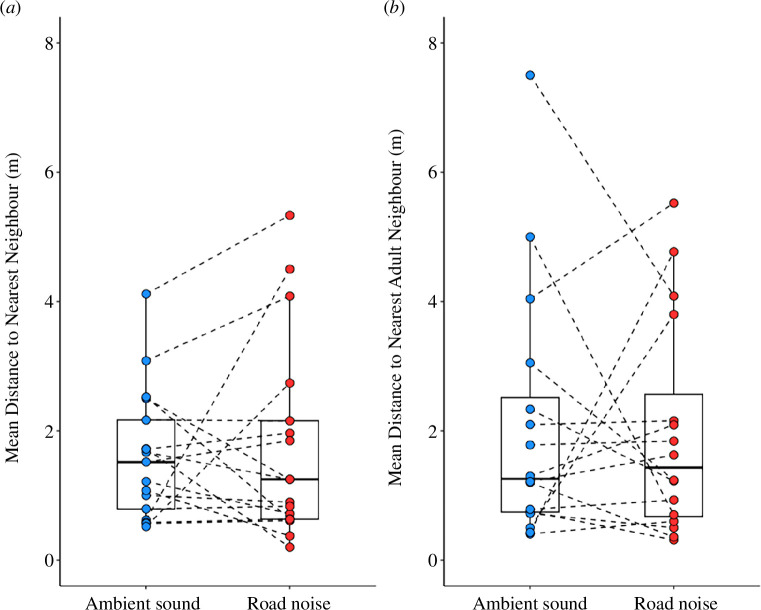
The impact of playback treatment (ambient sound or road noise) on the mean distance from the focal individual to (*a*) their nearest neighbour while foraging (*n* = 17) and (*b*) their nearest adult neighbour while foraging (*n* = 16). Boxplots indicate median and quartile values, whiskers represent data within quartiles ±1.5 times the interquartile range. Values for each trial are plotted separately, with dashed lines connecting paired trials completed on the same focal individual. Data points with the same value may overlap.

The immediate response (none or look-up) of focal dwarf mongoose pups and juveniles to playback initiation was not significantly affected by playback treatment (McNemar test: *X^2^* = 0.250, *p* = 0.617). From pre-trial to the first 10 s of the trial, playback treatment also had no significant effect on whether there was a change in nearest-neighbour ID (*X^2^* = 0.167, *p* = 0.683). When assessing all nearest neighbours, there was no significant effect of playback treatment (LMM: *t* = 0.250, *p* = 0.507) nor the interaction between age and playback treatment (*t =* 0.652, *p* = 0.545; table 1*a*; figure 1*a*) on mean nearest-neighbour distance. When assessing adult-only nearest neighbours, there was a significant effect of neither playback treatment (LMM: *t* = 0.507, *p* = 0.620) nor the interaction between age and playback treatment (*t* = −0.816, *p* = 0.428) on mean nearest-neighbour distance (table 1*b*; figure 1*b*).

**Table 1.** Output from linear mixed-effects model with a Gaussian error distribution and Wishart covariance prior specified, investigating the impact of road noise on the mean distance from the focal dwarf mongoose individual to (*a*) their nearest neighbour while foraging (*n* = 17) and (*b*) their nearest adult neighbour while foraging (*n* = 16); the difference in sample size attributable to one trial consisting of only non-adult nearest neighbours. Models included playback treatment (ambient, road), age (pup, juvenile) and their interaction as fixed terms, with focal individual identity (ID) nested within group membership included as a random effect. Variance (±s.d.) for the random terms (in italics) is reported.

fixed effect	effect ± s.e.	*t*	*p*
***(a) mean distance to nearest neighbour***			
intercept	0.977 ± 1.025	0.952	
treatment	0.105 ± 0.421	0.250	0.507
focal individual age	0.832 ± 0.700	1.188	0.335
treatment:age	0.377 ± 0.578	0.652	0.535
*ID:group*	1.053 ± 1.026		
*group*	2.299 ± 1.516		
***(b) mean distance to nearest adult neighbour***			
intercept	1.181 ± 0.684	1.725	
treatment	0.414 ± 0.816	0.507	0.620
focal individual age	1.570 ± 0.898	1.748	0.092
treatment:age	−0.887 ± 1.088	−0.816	0.428
*ID:group*	0.822 ± 0.907		
*group*	0.037 ± 0.192		

**Figure 2.**
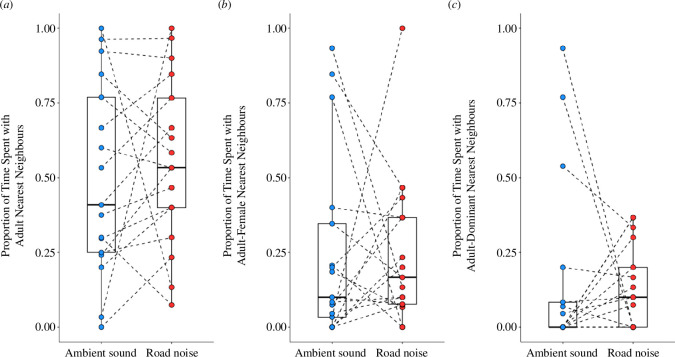
The impact of playback treatment (ambient sound or road noise) on the proportion of time that the focal offspring spent nearest conspecific neighbours who were (*a*) adults, (*b*) adult females and (*c*) dominant adults. Each panel contains a boxplot with median and quartiles, whiskers representing data within quartiles ±1.5 times the interquartile range. Values for each trial are plotted separately, with dashed lines connecting paired trials completed on the same focal individual. Data points with the same value may overlap (*n* = 17).

Similarly, we found that there was no significant effect of playback treatment on the age class, sex or dominance status of the nearest neighbour. There was no significant difference between ambient and road playback trials in the proportion of time that the nearest neighbour to a focal individual was (i) an adult as opposed to a non-adult (*z =* –0.096, *p* = 0.923), (ii) an adult female as opposed to an adult male (*z* = 0.148, *p* = 0.883) or (iii) a dominant individual as opposed to a subordinate (*z* = 0.082, *p* = 0.934), and this did not vary with the age of focal individuals (all age × treatment interaction terms *p* > 0.1; table 2; figure 2).

**Table 2.** Output from generalized linear mixed-effects models with beta error distributions and logit link functions investigating the proportion of time that focal offspring spent with nearest neighbours who were (*a*) adults, (*b*) adult females and (*c*) dominant adults. All models included playback treatment (road, ambient), age (pup, juvenile) and their interaction as fixed terms, with focal individual identity (ID) nested within group membership included as a random effect. Variance (±s.d.) for the random terms (in italics) is reported (*n* = 17).

fixed effect	effect ± s.e.	*z*	*p*
***(a) proportion of nearest neighbours that were adults***			
intercept	0.032 ± 0.425	0.075	
treatment	−0.056 ± 0.575	−0.096	0.923
focal individual age	−0.011 ± 0.619	−0.018	0.986
treatment:age	0.287 ± 0.805	0.356	0.722
*ID:group*	0.434 ± 0.659		
*group*	<0.001 ± <0.001		
***(b) proportion of nearest neighbours that were adult females***			
intercept	−1.005 ± 0.455	−2.208	
treatment	0.092 ± 0.623	0.148	0.883
focal individual age	−0.214 ± 0.613	−0.349	0.727
treatment:age	0.730 ± 0.916	0.797	0.426
*ID:group*	0.017 ± 0.129		
*group*	<0.001 ± <0.001		
***(c) proportion of nearest neighbours that were dominant adults***			
intercept	−1.927 ± 0.528	−3.654	
treatment	0.049 ± 0.590	0.082	0.934
focal individual age	−0.283 ± 0.545	−0.520	0.603
treatment:age	0.513 ± 0.776	0.661	0.509
*ID:group*	<0.001 ± <0.001		
*group*	0.059 ± 0.242		

## 4. Discussion

We found that dwarf mongoose offspring nearest-neighbour decision-making while foraging did not significantly differ when experiencing playback of anthropogenic noise compared with that of ambient sound. This lack of an effect was apparent in the immediate response to playback, alterations to nearest-neighbour identity (NNID) within the first 10 s of the trial, mean nearest-neighbour distance (NND) and nearest-neighbour intrinsic characteristics (age, sex and dominance status). Additionally, these responses did not significantly vary with the age of the focal individual. These findings contrast our predictions and the growing body of evidence demonstrating the detrimental consequences of noise on wildlife [1,5–7].

Noise induces a physiological stress response in an array of taxa [62–74], and elevated stress hormone levels can mobilize energetic resources for faster predator-avoidance responses, such as fleeing [75]. We, therefore, predicted that the potential increase in noise-induced stress would lead to a reduction in NND and changes to NNID to enable closer proximity to adult and dominant conspecifics for improved stress alleviation. Conspecific presence and proximity reduce cortisol levels and personal vigilance in other mammals, likely owing to collective predator-avoidance efforts [76–78], and previous work on dwarf mongooses found dominants to be more reliable sentinels compared with subordinates, resulting in reduced personal vigilance of foragers [24]. Yet, repeated exposure to a stimulus can result in a lessened response owing to gradual desensitization, increased tolerance or habituation [79,80], with experimental evidence that wildlife can habituate to chronic noise [81–83]. While our playback trials are unlikely to have provided sufficient noise exposure for habituation, the study population inhabits a reserve situated along a busy road. Thus, if habituation meant that there was no noise-induced stress from our road-noise playback, stress alleviation measures may not be required. Future research should explore the responses to chronic noise exposure, including how variation in those levels (e.g. for mongoose groups living different distances from the main road) might affect response changes over time, as this may provide insight into the capacity of the species for habituation.

Even if the study population had habituated to road noise, there is still the potential for masking of acoustic communication. Masking decreases the active space in which a signal can be transmitted [84,85], hence reducing the transmission distance between signaller and receiver could lessen masking. Yet, we found no evidence for a compensatory reduction in NND during road-noise playback. The dwarf mongooses may be compensating for communication masking using strategies other than a reduction in NND. For instance, many species alter the amplitude, frequency or timing of vocalizations to reduce the effect of masking by anthropogenic noise [86,87]. Compensatory strategies may not be limited to vocalizations; impaired acoustic communication can elicit a shift to alternative sensory modalities for communication [85,88,89]. Dwarf mongooses may be shifting communication ‘channel’ to rely more heavily on visual signals when acoustic signals are compromised by noise, substantiated by the increase in personal vigilance behaviour elicited in the study population by road-noise playbacks [34]. Yet, at the time of year the current study was conducted, dense vegetation frequently obscures lines of sight, meaning visual channels may also be compromised. Alternatively, if our study population has not habituated to noise, the absence of any alteration to NND could be attributable to the detrimental effect of noise-induced stress and distraction on decision-making ability. Recent research has similarly attributed deleterious decision-making to noise-induced stress and distraction [90–92]. A holistic approach when investigating behaviour under conditions of noise, in which multiple sensory modalities are accounted for, may provide explanation for the lack of change in NND under conditions of noise.

In principle, the absence of a difference in response to the two playback treatments in our experiment could occur if road noise falls outside of the acoustic signal-reception range of dwarf mongoose offspring; age-dependent variation in hearing ability has been documented across multiple taxa [93,94]. However, previous findings demonstrate dwarf mongoose offspring are more likely to flee upon initiation of noise playback compared with adults [34], rendering it unlikely that road noise is unheard by offspring. Alternatively, foraging in closer proximity to group members may be associated with increased competition over resources, such as prey items, and so intragroup competition—which is known to affect neighbour proximity and foraging-patch occupancy in other species [95–97]—could be mediating NND in dwarf mongooses. If the potential energetic costs and increased risk of injury associated with intragroup conflict over resources outweigh the benefits of alleviation from communication masking and stress, then this may prevent the reduction in NND in conditions of noise which we predicted.

There is ever-mounting evidence of maladaptive behavioural changes induced by anthropogenic noise [2,7], but there are also studies exhibiting no clear-cut effect of noise [98,99]. For robust conclusions to be drawn about the impacts of noise on dwarf mongoose offspring, experiments should consider alternative behavioural strategies being used to compensate for noise, using multiple response metrics (such as changes in vigilance behaviour and acoustic vocalization amplitude, frequency and timing). This is because assessing individual response metrics in isolation may omit interactions between multiple sensory modalities. Incorporation of the foraging rates of offspring, as well as adult provisioning behaviour to pups (none of which was seen during our relatively short experimental trials), may also provide a more representative indication of the decision-making of both adults and offspring in conditions of noise.

## Data Availability

All data and code can be accessed within electronic supplementary material files [100].
